# How gender norms and ‘good girl’ notions prevent adolescent girls and young women from engaging with PrEP: qualitative insights from Zimbabwe

**DOI:** 10.1186/s12905-022-01928-2

**Published:** 2022-08-16

**Authors:** Morten Skovdal, Camilla Lysemose Clausen, Phyllis Magoge-Mandizvidza, Freedom Dzamatira, Rufurwokuda Maswera, Rangarirayi Primrose Nyamwanza, Constance Nyamukapa, Ranjeeta Thomas, Simon Gregson

**Affiliations:** 1 Department of Public Health, University of Copenhagen, Øster Farimagsgade 5, 1014 Copenhagen, Denmark; 2 Manicaland Centre for Public Health Research, Biomedical Research and Training Institute, Harare, Zimbabwe; 3 Department of Infectious Disease Epidemiology, Imperial College London, London, UK; 4 Department of Health Policy, London School of Economics and Political Science, London, UK

**Keywords:** HIV, Prevention, PrEP, AGYW, Health services, Zimbabwe

## Abstract

**Background:**

Pre-exposure prophylaxis, or PrEP, has been hailed for its promise to provide women with user-control. However, gender-specific challenges undermining PrEP use are beginning to emerge. We explore the role of gender norms in shaping adolescent girls and young women’s (AGYW) engagement with PrEP.

**Methods:**

We draw on qualitative data from 12 individual interviews and three focus group discussions with AGYW from eastern Zimbabwe. Interviews were transcribed and thematically coded in NVivo 12. Emerging themes were further investigated using Connell’s notion of ‘emphasised femininity’.

**Results:**

Participants alluded to the patriarchal society they are part of, with ‘good girl’ notions subjecting them to direct and indirect social control. These controls manifest themselves through the anticipation of intersecting sexuality- and PrEP-related stigmas, discouraging AGYW from engaging with PrEP. AGYW recounted the need for permission to engage with PrEP, forcing them to consider engaging with PrEP in secrecy. In addition, limited privacy at home, and fear of disclosure of their health clinic visits, further heightened their fear of engaging with PrEP. PrEP is not simply a user-controlled HIV prevention method, but deeply entrenched within public gender orders.

**Conclusion:**

AGYW face significant limitations in their autonomy to initiate and engage with PrEP. Those considering PrEP face the dilemma of Scylla and Charybdis: The social risks of stigmatisation or risks of HIV acquisition. Efforts to make PrEP available must form part of a combination of social and structural interventions that challenge harmful gender norms.

**Supplementary Information:**

The online version contains supplementary material available at 10.1186/s12905-022-01928-2.

## Introduction

Adolescent girls and young women (AGYW) in sub-Saharan Africa are at heightened risk of acquiring HIV. Recent estimates suggest that six out of seven new infections amongst 15–19 year-olds in sub-Saharan Africa are among girls [1]. A number of risk factors have been found to fuel the HIV epidemic amongst AGYW, including early sexual debut [2], engagement in sexual relationships with older men [3], transactional sex [4], coerced sex [5], concurrent and overlapping sexual partners [6], and intimate partner violence [7]. School closures as a result of the COVID-19 pandemic have only amplified many of these risk factors [1]. Combined, these factors suggest that AGYW’s risk of contracting HIV is linked to their social position and limited control regarding decisions on when to have sex and use of contraceptives or HIV prevention methods [8]. HIV prevention methods like Pre-exposure Prophylaxis (PrEP), an antiretroviral pill, which women can take daily without the involvement of their male partners, is therefore of great priority [9].

However, whilst several clinical trials have demonstrated PrEP’s effectiveness in HIV prevention amongst several high-risk populations [10], including men who have sex with men [11, 12], intravenous drug users [13], and HIV-1-serodiscordant heterosexual couples [14], evidence about its effectiveness amongst AGYW trial participants in sub-Saharan Africa is less convincing [15, 16]. Uptake and successful engagement with PrEP by AGYW in sub-Saharan African does not seem to be undermined by lack of interest. On the contrary, acceptability studies from different sub-Saharan African countries indicate an understanding of the potential for PrEP to help them reduce risks of HIV acquisition. A study in Malawi found young women to be interested in PrEP, as they recognise their personal HIV risk to factors such as having multiple sexual partners, and inconsistent condom use [17]. A study from Kenya and Uganda found PrEP to be perceived as a means to help young women control their HIV risk in a context where they had limited control of male partners’ risky behavior [18]. Similar observations have been made in Uganda, where AGYW engaged in transactional sex saw PrEP as a tool to charge clients more for sex without a condom [19].

Nonetheless, in practice, many AGYW face hurdles to their successful engagement with PrEP. Qualitative studies have found HIV and PrEP-related stigma, scepticism towards the use of HIV drugs for prevention, fear of negative reactions from healthcare workers [20, 21] and lack of support from relatives, friends and partners, all contribute to low uptake of PrEP [22–24]. The role of significant others in shaping AGYW’s engagement with PrEP dominates the qualitative literature. Recent findings from the HPTN 082 PrEP study among AGYW in Harare, Zimbabwe, and Cape Town and Johannesburg, South Africa identify sexuality stigma and community-level PrEP stigma as key spheres of influence on PrEP disclosure and adherence [25]. Another study in Zimbabwe found AGYW decline or discontinue PrEP due to concerns linked to their partners finding out [26]. Relatedly, intimate partner violence has been associated with poor adherence to PrEP [27]. Whilst a growing number of studies allude to the impact of AGYW’s social position on their engagement with PrEP, little has been done to look at gender norms and PrEP use through the lens of social control mechanisms and “good girl” expectations.

This paper’s focus on gender norms is a direct reflection of our participants’ narratives, which highlight how traditional cultural gender role expectations interface with PrEP engagement. To help us understand and tell the story about how gender norms shape AGYW’s engagement with PrEP, we draw on Connell’s [28] notion of emphasized femininity. The concept acknowledges the unequal power distribution between men and women, and the subordination of women to men in a patriarchal gender order. Connell defines emphasized femininity as “compliance to this subordination and is oriented to accommodating the interests and desires of men” [28], p. 183]. Women may thus be subjected to an unequal distribution of power and expected to comply with this asymmetry. Connell [28] goes on to argue that this form of socially constructed femininity generally controls thoughts, behaviors, and beliefs. For the AGYW in our study, this may be exhibited through gender specific expectations of what it means to be a ‘good girl’, keeping them in roles demanding a down-prioritization of their own desires and needs [29], such as wanting to protect themselves from HIV and engaging with PrEP. Whilst there may be culturally dominant expectations of what it means to be a ‘good girl/woman’, femininity is clearly not one thing and may take many forms, and Connell [28] recognizes that expressions of femininity are defined by a complex combination of compliance, resistance and cooperation. Within this nexus we investigate how AGYW navigate, or balance, the social reward of complying with emphasized femininity, or ‘good girl notions, on the one hand, and the risks of being sanctioned through local morals, if adopting femininities based on resistance or non-compliance [30].

## Methods

The qualitative data presented in this paper are part a larger intervention study, which draws on multiple methods (qualitative, quantitative) and approaches (behavioural economics, community psychology) to understand and improve the uptake of HIV prevention methods, including PrEP, among young people in Manicaland, Zimbabwe [31]. The study obtained ethical approvals from the Medical Research Council of Zimbabwe (REF: MRCZ/A/2243), the institutional review board of the Biomedical Research and Training Institute in Zimbabwe (REF: AP140/2017), and the Imperial College London Research Ethics Committee (REF: 17IC4160). Written informed consent was obtained from all participants with the agreement that their identities would be kept confidential. Pseudonyms have been used throughout this study.

### Study location and participants

The qualitative methods were applied in two communities, which we call Saksom and Watku. Saksom is a high-density urban suburb, while Watku is a rural village and community. They were randomly selected due to their urban/rural location. Both are characterised by high levels of poverty and HIV. The HIV prevalence in Saksom and Watku is estimated to be in the range of 12.5 and 15%. Manicaland province is characterised by significant gender differences in HIV incidence, reaching nearly 1% for females and 0.5% for males [3]. Schaefer et al. [3] attribute AGYW’s increased risk of HIV acquisition most directly to age-disparate sexual relationships, and limited condom use. With AGYW being a core target group of the intervention study, we recruited 26 AGYW from the two communities to participate in in-depth individual interviews (IDIs) and focus group discussions (FGDs). They were purposefully sampled from a baseline survey, if they met all of the following eligibility criteria: between the ages 18–24, HIV-negative, sexually active, and considered “at risk” (according to a draft WHO risk screening tool), agreed to be contactable and volunteered to participate in the qualitative study. AGYW who met the criteria were contacted via telephone and invited to participate, none of those contacted declined to participate. Following advice from community consultations, participants received two bars of soap as a token of appreciation for their time. Whilst the broader study also interviewed community members, young men, parents and healthcare providers, this paper only reports on the perspectives of AGYW. The group turned out to be very diverse. Both married and unmarried AGYW were represented, some had one or more children while others had none. Most had gone to secondary school and finished form 3 or 4. Some were, at the time of the interview, studying at college, whilst others worked by buying and selling goods. Many of those who were married stayed at home, taking care of the family. Whilst some had established their own family, others stayed with relatives such as parents, grandparents, or siblings. About three quarters of participants had heard of PrEP, but their knowledge was limited. Interviewers therefore provided a brief introduction to PrEP prior to the interview. None of the participants disclosed PrEP use, but a few had friends who had just taken up PrEP.

### Data collection and analysis

A total of six IDIs and two FGDs consisting of four and five participants were conducted in Saksom (n = 15), whilst six IDIs and one FGD with five participants were conducted in Watku (n = 11). Data were generated between March and June 2019 by two qualitative researchers who spoke the local Shona language. The researchers were thoroughly trained in study objectives and procedures. The researchers drew on semi-structured topic guides, covering themes such as experiences of being an AGYW, relationships and sexuality, as well as their perspectives on HIV risk practices, and prevention technologies, such as PrEP. Whilst the FGD and IDI topic guides shared empirical objectives and covered the same themes, the IDI topic guide included several concrete questions under each theme (see Additional file 1). Questions focused on lived experiences, attitudes, structures and practices affecting AGYW uptake of PrEP. No questions were asked directly about gender norms, or ‘good girl’ notions. The FGD topic guide presented discussion topics, with the intent of generating data through group interaction. On average, the IDIs lasted 53 min, while the FGDs lasted an average of 80 min. The locations for the IDIs were chosen by the participants themselves. IDIs were typically conducted either inside or nearby the participant’s homes, or in some cases at the participant’s shop. FGD were conducted in nearby classrooms, community halls, or in a project room of a local organization. Whilst efforts were made to keep the interview spaces free from interruption, a few IDIs were temporarily interrupted by small children or other individuals entering.

All IDIs and FGDs were digitally recorded, transcribed, and translated into English. Protocols were in place to double check the quality of transcription and translation. Transcripts were subsequently imported into NVivo 12 for thematic coding and analysis. This process followed the thematic network analysis steps outlined by Attride-Stirling [32]. Her technique systematically organizes and indexes qualitative material into a web-like network of themes that unfold a story by moving from text to interpretation. Practically, this involved reading the transcripts thoroughly, while noting ideas, thoughts, and impressions. Codes and themes were initially generated inductively. This involved the second author, CLC, assigning descriptive codes to text segments within the transcripts. As gender norms emerged as a key factor shaping AGYW’s engagement with PrEP, a conceptual framework was devised collaboratively by the first author, MS, and CLC in conversation with the emerging themes. This led to a refining of codes and themes by merging, renaming, and splitting existing codes before clustering them into more deductively derived themes from which key gendered spheres of influence emerged. Through collaborative efforts, a total of 11 basic themes, four organizing themes and a global theme were generated. The basic themes are direct representations of participant accounts, whilst the organising themes and global theme constitute further levels of abstraction and interpretation, aided by Connell’s notion of ‘emphasised femininity’. The themes are outlined in Table 1 and form the structure of our results section.

**Table 1:** Thematic network: from basic themes to global theme

Basic themes	Organising themes	Global theme
Patriarchy and poverty	1. AGYW in a man’s world	Gendered spheres of influence, underpinned by ‘good girl’ notions preventing AGYW from engaging with PrEP
HIV risk		
Taboos surrounding pre-marital sex	2. Stigmatisation as a gender-control mechanism affecting AGYW PrEP use	
PrEP-related stigma		
PrEP signals infidelity		
Need permission to engage with PrEP	3. AGYW’s limited freedom of choice in PrEP use	
Need permission to go to health clinic		
Lack of privacy at health clinics	4. The struggles of AGYW to keep PrEP use hidden	
Perceived healthcare provider attitudes		
Lack of nurse confidentiality		
Limited privacy at home		

## Results

A prominent representation of emphasized femininity and imbalanced power relations between men and women dominated participants’ accounts of their social reality vis-a-vis opportunities to engage with PrEP. Even though some participants distanced themselves from notions of being suppressed by what we in this paper refer to as emphasized femininity (cf Connell [28]), for instance by expressing views of sexual confidence and independence, it was a consistent reference point throughout the data.

### AGYW in a man’s world

By way of introduction to the context, most participants left little doubt that they feel they live in a male dominated world where women are subordinate to men. All participants made references to existing norms diminishing their voices. Men were talked about as “head of house” and the one “making all the important decisions.” Men were said to have the “upper hand”, whereas women “will have to listen”. This patriarchy was noted to limit young women’s sexual decision-making. It was mentioned that being a woman means that you have to “sexually satisfy your husband” and “it’s a matter of being controlled by the man”. The general impression was that “in this community, most men love women too much”, and AGYW therefore had to negotiate their sexuality in a context where they feel exposed to extensive attention and superiority of men. Many participants explained that it is ‘normal’ for men to have many sexual partners while women are expected to stay faithful. Though this formed the basis for a general mistrust towards men and raised concern among some participants, men’s infidelity was generally expected. One married participant explained:

“[..] if you have a husband, you shouldn’t really trust that he is faithful [..] because you don’t know what he does when you are not around [..] you are supposed to expect anything from him, even getting infected with HIV is possible.” Jane, Saksom, IDI

Unbalanced dynamics also appeared in explanations of why sexual encounters between women and men are initiated. Many participants said that men most often engage in relations for sex, while AGYW often expect marriage or financial returns. During a FGD, one participant, Sabrina, explained:

“The ways that I end up sourcing out for money are the ones which will make me become susceptible to HIV.” Sabrina, Saksom, FGD

Patriarchy and scarce resources are contextual factors which were said to increase AGYW’s risk of acquiring HIV. However, interesting differences in risk perception emerged from our analysis. Unmarried participants rarely considered themselves at risk of acquiring HIV. Starting out by laughing at the question on whether she saw herself to be at risk for HIV, one participant, Mary, an IDI participant from Saksom said: “no I am not at risk. […] I am protecting myself.”—projecting a resistance to the emphasised femininity otherwise represented in the interview material. Married participants on the other hand, like Jane, did acknowledge their own risk of getting infected, attributing this to their husbands’ risky sexual behaviors. They presumed that their husband’s extramarital affairs would leave them at risk of HIV.

The patriarchal and social realities described above provide context to the form of emphasised femininity characterising the lives of AGYW in this setting. It not only puts them at risk of acquiring HIV, but, as we will now demonstrate, challenges their engagement with PrEP as a means of protecting themselves.

### Stigmatisation as a gender-control mechanism affecting AGYW’s PrEP use

PrEP is revealing of a sexuality. However, in a context where women’s sexuality is ascribed to men’s desires and demands, and where pre-marital sex is considered immoral, AGYW’s engagement with PrEP is challenged by fear of judgement and stigmatising attitudes. Several of the unmarried participants expressed that they would feel ashamed taking up PrEP as they were “not expected to be sexually active”. Participants explained that even if they were motivated to engage with PrEP, anticipating sexuality stigma would prevent them from doing so:

“I think what will make her not use [PrEP] is […] the stigma that is attached to young women who go looking for HIV prevention methods because that easily tells the community that you are sexually active.” Rose, Saksom, FGD

“They [her parents] will most likely be against it [PrEP] […] They will think I was being [sexually] mischievous.” Mary, Saksom, IDI

PrEP was thus perceived to violate expectations of what it means to be ‘a good girl’. It signals taking control of their sexuality, which they are not entitled to if ideal femininity ideologies are to be upheld [30]. That said, a few participants expressed resistance to this emphasised femininity by describing how they take control over their sexuality:

“Women are the ones who are shy [but] Personally I do not face any problems […] I am able to protect myself. I use condoms and I can collect condoms whenever I want. […] I will also seek out PrEP.” Mary, Saksom, IDI

For many of our participants, these representations of what it means to be ‘a good girl’ came from parents. Many of the unmarried participants explained that their parents would be disappointed if they found out they were having sexual relations before marriage. One participant said:

“I can also add the issue of parents because they are the ones who put pressure on us. If they discourage us to take PrEP, then it becomes difficult for us since we will be trying to be good children and obedient children who listen to our fathers and mothers.” Charlotte, Saksom, FGD

A number of participants in relationships spoke about how they would most likely encounter enacted stigma from their male partners if their use of PrEP were discovered. They explained that their male partners not only perceived girls’ PrEP use as a sign of mistrust to the partner but also a sign of AGYW’s own infidelity. One of the married participants said:

“[…] he will say that you are thinking of cheating on me or being unfaithful to me that’s why you want to access PrEP.” Monica, Watku, IDI

Our participants, while considering PrEP, have to balance their perceived risk of contracting HIV with the social risk of their parents’ or partners’ judgement and disappointment. However, this form of anticipated sexuality stigma was not the only form of stigma preventing AGYW from engaging with PrEP. Different stereotypes surrounding AGYW on PrEP also emerged from the interviews. Women in sex work were the first to be targeted with PrEP in Zimbabwe [33] and several participants said that besides being a sign of sexual activity, PrEP may also signal promiscuity and carried associations with being a woman engaged in transactional sex. Several participants were thus concerned about being judged and labelled as “loose woman” if they sought out PrEP, as explained by one participant:

“Imagine being seen going to get PrEP just before your husband comes back. The assumption will be that you are a prostitute and you were sleeping around in your husband’s absence. If you are a girl who is not married they will say you sleep around or you are the lady of the night [and] maybe you just have one partner but you are not trusting them so you have to protect yourself.” Claire, Saksom, IDI

Arguably, the anticipated stigmas outlined above affect AGYW’s decision-making regarding PrEP and constitute social control mechanisms. These mechanisms are part of, and further entrench the dominant form of emphasised femininity and gendered expectations that dominate in this cultural setting.

### AGYW’s limited freedom of choice in PrEP use

PrEP has been hailed as a user-controlled and female-controlled HIV prevention method, allowing AGYW to protect themselves from HIV without having to negotiate safe sex practices at the time of sex. However, whilst anticipated stigmas constitute more hidden and indirect forms of control, our data also reveal more direct forms of control, highlighting limitations to women’s autonomy to initiate and engage with PrEP.

Whilst some participants did indeed indicate that PrEP would allow them to protect themselves from HIV without their partner’s involvement, others said that PrEP use, or at least consistent usage, would be impossible without their partner’s or parent’s permission. A couple of participants, who considered themselves at risk due to expectations of their husbands’ extramarital affairs said:

“For me to use protection it is not possible. He will refuse. He will not allow me to take medication such as PrEP. He will not allow that. So, you will end up just saying whatever comes I will take it.” Jane, Saksom, IDI.

“[…] husbands […] will refuse [use of PrEP] and at the end you reach a dilemma because you can’t go against your husband’s decision.” Cameron, Watku, IDI

These quotes not only highlight the women’s subservient position—that engagement with PrEP requires permission, but also exemplifies their powerlessness and acceptance of their subservient position in the relationship, and the dilemmas this brings. Adding to this, some participants spoke about how their economic dependency on partners meant they did not have the means (e.g., bus fares) to visit the health clinic. Some unmarried AGYW also spoke about the need for permission, not from their sexual partners, but from their parents:

“I may be willing to use PrEP but my parents may discourage me or refuse for me to use it. There are not a lot of parents who will agree for their children to use PrEP, they don’t even want to hear about it.” Charlotte, Saksom, FGD

In a FGD in Watku, a conversation about taking PrEP secretly highlighted that this would be difficult, as many of the girls needed permission from either their partners or parents to go to the health clinic, despite not being a legal requirement. The consequence for accessing PrEP without permission was occasionally described using war metaphors, e.g., “it will raise wars at home”. A few FGD participants disagreed fully with this representation of parents and argued that some parents do indeed understand the importance of HIV prevention for their daughters and would welcome PrEP. Such conflicting accounts highlight important spaces and opportunities for change.

### The struggles of AGYW to keep PrEP use hidden

In principle, PrEP provides its users with the possibility of taking it privately without involving others. However, this privacy is challenged by the very same gender norms and forms of social control that require many AGYW to engage with PrEP in secret. Some participants feared going to the health clinic for accessing PrEP, as they may get recognised by members of their community. Several participants spoke about the indiscreet set up within health facilities, with visits exposing them to their broader community. In different FGDs, two participants explained:

“I mean a hospital set up is shared with adults and so the moment I go to access PrEP and the elders get to know about it. The moment adults know about it they will know that I am sexually active and inevitably this information will get to my parents, and it will be problematic because culturally I am not expected to be sexually active given that I am not yet married. So it is difficult to access it there.” Lisa, Saksom, FGD

“So other patients who have come to the clinic for their own business will see that you are carrying PrEP pills hence spreading the gospel to other community members that so and so is taking PrEP. So this problem may even make you file for divorce with your husband because he would have heard through grapevine that you are taking PrEP pills.” Carol, Watku, FGD

Fear of the perceived public nature of accessing PrEP was exacerbated by the organisation of PrEP delivery. In this particular context, PrEP was made available in departments for opportunistic infections at local hospitals and clinics, which are locally known for being the departments distributing antiretroviral drugs for people living with HIV. Some participants thus expressed a fear of being seen near this department, worrying people may think of them as HIV positive. Even if the PrEP pills were concealed, for instance in response to Carol’s concern, their entrance to, or exit from the Department may still subject the girls to stigma. Interviews also revealed that AGYW feared sharing private matters with nurses. They feared both their judgemental attitudes (related to the stigma theme discussed above) and their indiscretions. Many participants were convinced that healthcare providers would directly share private information with other community members or relatives.

“Nurses from the facility will have spread the gospel that I have undertook PrEP so by the time I get to Watku people will start calling me names and bashing me saying that I take PrEP in a stigmatizing way so that reduces the number of people who will go and access PrEP at the clinic.” Anna, Watku, IDI

“[…] the nurses won’t be secretive and confidential to the extent that when I get home others at home would be knowing already that I went to the local clinic to access PrEP. So these are some of the challenges that may even lead to divorce.” Carol, Watku, FGD

The quotes further remind us of the consequences (e.g., stigmatisation or divorce) that AGYW anticipate—as a form of social control—if their engagement with PrEP is revealed. Several participants also questioned the practicalities of taking PrEP at home without being discovered. They spoke about their limited privacy at home, and how this may complicate engagement with undisclosed PrEP use. During an interview, one participant reflected on the time where she was still living with her mother:

“If you check our parents, like my mother used to get into my room and move stuff around. At times you would come from school and then see your books all over the room meaning she would have turned the place upside down looking for something so where to put the PrEP pills is a challenge if you are a girl and not yet married.” Anna, Watku, IDI

Similar challenges with partners were noted. One participant explained that by living together, her partner would eventually find out.

“I may eventually decide to go and access PrEP, but when I want to take the pills at home it becomes a challenge because my husband will always be at home.” Carol, Watku, FGD.

Whilst many participants echoed these concerns, some showed a willingness to overcome these challenges and offered practical solutions:

Suzanne: “(laughs) and how would you take the pills silently?”

Emma: “If push comes to shove, you will take PrEP pills from the toilet or let’s say when you are taking a bath and then you go to the bathroom with your pills, you won’t face any problems at all.” Emma, Watku, FGD

## Discussion

We set out to explore the role of gender norms in shaping opportunities for AGYW to engage with PrEP. We found that in this cultural setting, persistent gender norms and notions of what it means to be a ‘good girl’ infused the responses of all our participants. Partners, parents, and healthcare workers were often said to ascribe to ‘good girl’ notions, which they sought to maintain through stigmatisation and social control mechanisms. The article demonstrates how these mechanisms come together in complex ways, with stigmas of sexuality, HIV prevention and PrEP being deeply intertwined through what Turan et al. [34] refer to as intersectional stigma. For instance, we found that many AGYW said they would struggle to engage with PrEP due to the notion that pre-marital sex among AGYW deviates from local understandings of a ‘good girl’ and the perception that PrEP signals sexual misbehavior and infidelity.

Many AGYW spoke about their disempowered social position, and the potential risks and consequences of engaging with PrEP. Men’s control over AGYW’s PrEP use, and the gender dynamics at play, has been observed in other studies as well. A study from Malawi found that AGYW perceive PrEP to be harmful to marital trust. Married AGYW, just like the participants in our study, believed their husbands would accuse them of having extramarital affairs if they were to discover their wives’ use of PrEP [17]. A study among pregnant AGYW in Kenya found that they feared their engagement with PrEP may result in violence from their partners [35]. Fearing the perceived consequences of PrEP use led many of our participants to talk about the need to seek permission to engage with PrEP from parents and partners. Similar observations have been made in rural Kenya and Uganda, where many AGYW reported that permission from partners to engage with PrEP may be inevitable [18]. As many of our participants anticipated disapproval of PrEP engagement by parents or partners, they went on to explain that they may be forced to either decline PrEP as an HIV prevention method or engage with it in hiding. However, this too many participants spoke about as being practically difficult and potentially dangerous. This resonates with observations made by Camlin et al. [18], who provide details of husbands locating PrEP pills and confronting their wives.

We found that the social control of AGYW’s sexuality spills into the community, underlining the presence of a collectively shared understanding of an emphasised femininity, or what it means to be ‘a good girl’. One space where this control became evident was at the health facility, a topic we discuss in detail elsewhere [20], elaborating on our participants’ concern about healthcare worker attitudes and indiscretions. Our observations chime with findings from Tanzania, where a study has found that healthcare workers who held negative attitudes toward adolescent sexuality were much less willing to provide PrEP to AGYW [36]. In agreement with calls made by women in sex work in South Africa [37], health clinics delivering PrEP need to be safe spaces, free of judgement from healthcare providers.

Whilst other PrEP studies have alluded to the impact of gender in passing, as far as we know, this is the first study to explore in depth how gender norms and ‘good girl’ notions come to influence the (in)ability of AGYW to engage with PrEP. Our study has highlighted the difficult dilemma of Scylla and Charybdis, which AGYW face when considering PrEP. On one hand they face multiple social risks of engaging with PrEP, such as stigmatisation, or even violence or abandonment. On the other hand, if they refrain from engaging with PrEP or other HIV prevention methods, they may be at increased risk of acquiring HIV. AGYW in this context thus must weigh their perceived risk of acquiring HIV against their perception of the social and physical risks imposed by their surroundings, whether at home with their parents or partners, or in the health clinic. At the moment, the social risks of engaging with PrEP appear to carry greater weight, contributing to low uptake and inconsistent engagement.

These findings are constrained by a few methodological limitations. First, our cross-sectional design and reliance on interview data may only have captured self-reported experiences and perspectives at one particular moment in time. Future research may consider adopting a more in-depth and longitudinal ethnographic approach, studying shifts in gendered norms and household dynamics over time through participant observation methods. Second, our study only reports on the hypothetical experiences and perspectives of AGYW. In future studies we will broaden the scope to include AGYW taking PrEP as well as the perspectives and experiences of parents, community members, healthcare workers and young men.

## Conclusions

AGYW in parts of eastern Zimbabwe, because of their gender and the cultural context within which they live, are exposed to a form of emphasised femininity, or ‘good girl’ expectations, which when enforced though stigmatisation and social control mechanisms, affect AGYW’s (in)ability to engage with PrEP. PrEP is not simply a ‘user-controlled’ HIV prevention technology. Its effectiveness is highly dependent on the complex social fabric that AGYW form part of. Initiatives to make PrEP available must therefore be implemented alongside a combination of social and structural interventions, and as this paper demonstrates, particularly those that challenge harmful gender norms, gender inequalities and gendered power imbalances. Our findings stress that such interventions must address the connections between different community-embedded determinants of preventive behaviours [38], something which is best achieved through community mobilisation activities in combination with broader policies and programming [39]. For instance, to break the connection between PrEP stigma and ‘good girl’ notions, gender-transformative community mobilisation activities must be implemented alongside PrEP programming that is more inclusive of the wider population, rather than women in sex work first then others, to reduce “establishing” stigma. Velloza et al. [25] have found community and clinic-based discussions about PrEP, adherence clubs and other activities normalizing sexual behaviour and PrEP use, can empower AGYW in their PrEP use and to cope with anticipated stigma.

## Supplementary Information

**Additional file 1:** Topic guide for individual interviews and Topic guide for focus group discussions.

## Data Availability

The interview and focus group guides are included in Additional file 1. Data used for the study are available from the corresponding author on reasonable request.

## Abbreviations

AGYW: Adolescent girls and young women
FGD: Focus group discussion
HIV: Human immunodeficiency virus
IDI: In-depth individual interview
PrEP: Pre-exposure prophylaxis
