# Supplementary material for: How gender norms and ‘good girl’ notions prevent adolescent girls and young women from engaging with PrEP: qualitative insights from Zimbabwe
Source: BMC Womens Health. 2022 Aug 16;22:344. doi: 10.1186/s12905-022-01928-2 (PMC9379870; doi:10.1186/s12905-022-01928-2)
Supplement: Supplementary file 1 — Additional file 1: Topic guide for individual interviews and Topic guide for focus group discussions. [file 12905_2022_1928_MOESM1_ESM.docx]

Additional file 1

**Topic guide for individual interviews**

| **QUESTIONS AND PROBES** | **NOTES/RATIONALE** |
| --- | --- |
| **Background/icebreakers** |  |
| ***I’d like to begin by asking a question about you:***   - Can you tell me a little bit about yourself? *Probe: age, marital status, children, work, income sources, education* | To understand what type of life the participant lives. You should bear these responses in mind during the rest of the interview. |
| **Being young in your community** |  |
| ***We are curious about what it is like to be a young person in your community, and will ask you a few questions about that:***   - Can you tell me what has it been like for you to grow up as a young person in this community? - How do parents in your community relate to their children? - What are the advantages or disadvantages to being a man or a woman? | To understand young people’s views on what it is like to be a young person in this community |
| **Relationships and sexuality** |  |
| ***We would like to hear about how relationships develop and have a few questions about sex:***   - How and when do young people in this community begin to form sexual relationships? Are they ready for it? - What would young people like to gain from a sexual relationship? Are there differences between young women and men? - How do you know if, or when, you can trust your sexual partner? - Can you tell us a little bit about your last relationship? What was its duration? how often did you see each other? how committed were you? How did you develop trust in the relationship? | To understand the young person’s experiences of, and perspectives on, relationships and sexuality |
| **HIV risk awareness** |  |
| ***We would like to hear about how HIV has affected young people’s relationships:***   - When you form a sexual relationship, are you concerned about HIV? - Do you consider yourself to be at risk of HIV? if not, why do you think that is the case? - Why do young people engage in activities that put them at risk of HIV? - Do young people in your community talk about sex and HIV risk with each other? - Do young women and men consider risk differently? How? Why do you think that is? | To understand young people’s awareness of HIV, their risk perception, and the implications of HIV on their relationships and sexuality |
| **HIV prevention behaviours in your community** |  |
| ***We’re interested in hearing about HIV prevention behaviours in your community:***   - What do you do to avoid HIV? - What are some of the challenges for young people, like yourself, to avoid HIV? - Are these challenges the same for young women and men? If not, please explain. | To understand what young people in the community do to avoid HIV |
| **HIV prevention options** |  |
| ***Imagine that 17-year-old ‘Zivai’ is in a sexual relationship with a 32-year-old man from her community. Zivai is aware of the HIV risk of sleeping with a man older and more experienced than herself. Zivai would like to ensure that she does not contract HIV.***   - What kinds of HIV/STI prevention methods do teenage women, like Zivai use? - Why would she use, or choose not use, HIV prevention methods like condoms/PrEP/partner testing? - If Zivai wanted to access HIV prevention methods or services, what methods or services would she be looking for? Where and how should such services be offered? - What factors would make it difficult for a person, like Zivai, to access HIV prevention methods? Why? How can these challenges be addressed? [probe: community norms, parents] | To understand the young person’s view on what prevention methods are available in the community and challenges in accessing and using these methods. |
| **Pre-exposure prophylaxis** |  |
| ***Let’s talk about PrEP:***   - Do you know what PrEP is? (If not, please explain to the participant) - What do you think about PrEP as a HIV prevention method? - How might PrEP be viewed in your community? What are your views on PrEP services being delivered in your community? - What do young women think about PrEP as a prevention method? What do you think about it? - What are the advantages of PrEP? Why do some young women want PrEP? - What are the limitations of PrEP? Why do some young women refuse to get PrEP? - What would convince these young women / you to access PrEP? - What community factors prevent young women from accessing PrEP? [probe: community norms, parents] - How would your parents react if you enrolled onto ART? - To whom would you disclose that you are on PrEP, if anyone? - If you were going to access PrEP, where would you like to access it? How should this service be delivered? - How can we address barriers to uptake of PrEP? | To understand what AGYW think about about PrEP in the community and challenges to service delivery |
| **Combination of HIV prevention methods** |  |
| ***The safest option is not to rely on one HIV prevention method, but to combine multiple methods. Now, let’s talk about combination HIV prevention:***   - What combination of steps do young people take to avoid HIV? - What factors prevent young people from using multiple HIV prevention methods at once? - What combination of steps do you take to avoid HIV? | To understand what AGYW think about about combination HIV prevention |
| **Recommendations** |  |
| ***Finally, I’d like to ask for your recommendations to help improve the uptake of HIV prevention services in this community.***   - What three things do you think should happen in this community to improve the uptake of HIV prevention methods amongst young people in this community? - What three things do you think should happen in this community to improve the uptake of HIV prevention methods amongst young people in this community? | To understand what AGYW feel need to change in the community and in health services. |
| **Wrap up** |  |
| ***Finally:***   - Is there anything else that you’d like to discuss today about your life or your experiences regarding access to, or us of, HIV prevention methods? - Are there any questions that you’d like to ask me about anything that we have discussed today? |  |

**Topic guide for focus group discussions**

| **QUESTIONS AND PROBES** | **NOTES/RATIONALE** |
| --- | --- |
| **Icebreaker: Being young in your community** |  |
| - Can you tell me what has it been like for you to grow up as a young person in this community? | To understand what it is like to be a young person in this community |
| **Relationships and sexuality** |  |
| - How and when do young people in this community begin to form sexual relationships? Are they ready for it? | To understand young people’s relationships and sexuality |
| **HIV risk awareness** |  |
| - What are the risks, if any, of being in a sexual relationship? - What young people are at risk of HIV? Do they know they are at risk? What is that the case? - Why do young people engage in activities that put them at risk of HIV? | To understand young people’s awareness of HIV, their risk perception, and the implications of HIV on their relationships and sexuality |
| **HIV prevention behaviours in your community** |  |
| ***We’re interested in hearing about HIV prevention behaviours in your community:***   - What do young people do to avoid HIV? - What are some of the challenges for young people to avoid HIV? | To understand what young people in the community do to avoid HIV |
| **HIV prevention options** |  |
| ***Imagine that 17-year-old ‘Zivai’ is in a sexual relationship with a 32-year-old man from her community. Zivai is aware of the HIV risk of sleeping with a man older and more experienced than herself. Zivai would like to ensure that she does not contract HIV.***   - What kinds of HIV/STI prevention methods do teenage women, like Zivai use? - Why would she use, or choose not use, HIV prevention methods like condoms/PrEP/partner testing? - If Zivai wanted to access HIV prevention methods or services, what methods or services would she be looking for? Where and how should such services be offered? - What factors would make it difficult for a person, like Zivai, to access HIV prevention methods? Why? How can these challenges be addressed? [probe: community norms, parents] | To understand young people’s views on what prevention methods are available in the community and challenges in accessing and using these methods. |
| **Pre-exposure prophylaxis** |  |
| ***Let’s talk about PrEP:***   - What do you think about PrEP as a HIV prevention method? - What factors would make it difficult for a young person to access PrEP? [probe: community norms, parents] | To understand what young people think about about PrEP in the community and challenges to service delivery |
| **Recommendations** |  |
| ***Finally, I’d like to ask for your recommendations to help improve the uptake of HIV prevention services in this community.***   - What three things do you think should happen in this community to improve the uptake of HIV prevention methods amongst young people in this community? | To understand what they feel are priorities for change in the community and in health services. |
| **Wrap up** |  |
| ***Finally:***   - Is there anything else that you’d like to discuss regarding access to, or us of, HIV prevention methods? - Are there any questions that you’d like to ask me about anything that we have discussed today? |  |
